# Supplementary material for: Vaccination conspiracy beliefs among social science & humanities and STEM educated people—An analysis of the mediation paths
Source: PLoS One. 2022 Mar 1;17(3):e0264722. doi: 10.1371/journal.pone.0264722 (PMC8887742; doi:10.1371/journal.pone.0264722)
Supplement: S1 Highlights — (DOCX) [file pone.0264722.s002.docx]

Highlights

1. Vaccination conspiracy beliefs more present among respondents educated in social science & humanities then STEM

2. Indirect effect of the differences in science literacy confirmed

3. Lower level of science literacy, belief in natural immunity and institutional distrust confirmed as predictors of vaccination conspiracy beliefs

4. Positive correlation between religiosity and vaccination conspiracy beliefs

5. Vaccine conspiracy beliefs more present among women than men
