# Supplementary material for: Vaccination conspiracy beliefs among social science & humanities and STEM educated people—An analysis of the mediation paths
Source: PLoS One. 2022 Mar 1;17(3):e0264722. doi: 10.1371/journal.pone.0264722 (PMC8887742; doi:10.1371/journal.pone.0264722)
Supplement: Attachment — Submitted filename: RECENZIJA Plos.docx [file pone.0264722.s003.docx]

Review

**Manuscript Number PONE-D-21-19906**

**"Vaccination conspiracy beliefs: differences between persons educated in different scientific fields and their mediating mechanisms"**

This article is exploring the differences in vaccination conspiracy beliefs between people belonging to different scientific fields – Social Sciences & Humanities (SH) and Science, Technology, Engineering and Mathematics (STEM). The results revealed that those educated in SH were more prone to vaccination conspiracy beliefs, and this was mediated by health beliefs; lower trust in the healthcare system, and lower science literacy.

Although the authors deal with important questions for the society today, the rationale for this specific research question is not explored enough. The theoretical background need to be more explained, followed by the importance of differentiation between SH and STEM persons. Is there any specific recommendation coming from these findings (in terms of science education)?

Some issues remained unanswered: What kind of professions exactly, do the authors have in those groups under the umbrella of STEM and SH? What are the employment statuses of the respondents? Sometimes people work in completely different fields then those from which they graduated. It would be good to discuss the relation between employment-education status of the respondents and relation of those variables to the conspiracy belief.

Structure of the manuscript in some parts is not adequate for the academic paper. There is no difference between Introduction and Method. Research goals and questions should be placed in the Introduction part. Method part is missing; it should stand alone not incorporated in other parts. Instruments should be placed in the Method part. Sample information is not sufficient: we need to know when the survey was conducted, whether there was ethical approval from any institution, the length of the questionnaire. Also I would recommend reporting the sample characteristics in more details.

Additionally, descriptive statistics are usually part of results not method part. Endorsement of scientific knowledge and vaccine conspiracy belief should be mentioned as research question, and the total scores could be given separately for SH and STEM part

Tables are not according to APA standards.

I would also like to see empirical confirmation for the assumptions mentioned in the discussion: *Additionally, a large portion of these differences can be explained by the mediating factors that are part of the 3C's model which, according to our claim, might be stemming from the differences in epistemic cultures, i.e. teaching patterns, cognitive approaches, research traditions, goals, behaviours, logic and methods.* How can authors assume that? In the discussion part it would be better if the authors analyse results that they measured in the study.

Finally, what is the relation between Conspiracy beliefs vs. Vaccine intentions? What are the effects of conspiracy belief in the current pandemic? And how can the results of the study be connected to the situation in pandemic – covid-19 conspiracy theories.
