# Supplementary material for: Vaccination conspiracy beliefs among social science & humanities and STEM educated people—An analysis of the mediation paths
Source: PLoS One. 2022 Mar 1;17(3):e0264722. doi: 10.1371/journal.pone.0264722 (PMC8887742; doi:10.1371/journal.pone.0264722)
Supplement: Attachment — Submitted filename: Response to reviewers.docx [file pone.0264722.s004.docx]

Response to Editor/Reviewers

Dear Editor,

Dear Reviewers,

Thank you for giving us the opportunity to revise and resubmit this manuscript. We appreciate your suggestions and we have incorporated them into the revised manuscript. We hope that paper is significantly improved in this way. All our specific responses are highlighted in blue.

Reviewer #1: Thank you for the opportunity to review the manuscript entitled ‘Vaccination conspiracy beliefs: differences between persons educated in different scientific fields and their mediating mechanisms'.
The author(s) present a study in which they investigated vaccine conspiracy beliefs in regard to educational background – social science and humanities versus stem. It is an interesting take on the role of education in vaccine conspiracy beliefs, because education is in this kind of research mostly investigated in terms of educational levels and not field differences.
I found the manuscript to be well written, and it contributes with new insights. Nevertheless, I recommend some changes which can contribute to an even greater quality of it.

Title
I suggest renaming the title to emphasize your main finding and make it more interesting for potential readers, some examples are: ‘Vaccine conspiracy beliefs stronger in social sciences and humanities then in STEM – a correlational study’.

Reply: We changed a title to make it clearer, and to emhasize our findings. Thank you for the suggestion.

Abstract
Start with an introduction sentence.

Reply: Added.

Introduction
Page 4, lines 76-79, add references for describing the SH and STEM fields.

Reply: Done.

Throughout the manuscript replace ‘persons’, perhaps with ‘people’, although I am not sure this is the adequate term too.

Reply: Where appropriate (in case where we are talking about the persons who participated in the survey), we changed „persons“ into „participants“. In other cases, we changed „persons“ into „people“.

Research goals, questions, and methods
Please insert a paragraph that in details describes the procedure of how and when the study was conducted - where was the survey shared, which months and year, how the instruments were translated to Croatian.

Reply: we added the requested details, and also explained that the data were taken from the wider pilot study of vaccine hesitancy in Croatia.

Check the journal guidelines – but I find the Descriptive tables should be better put in the Results section.

Reply: We understand your point, but we kept it in the Methods (now: Methods and instruments) section since they describe the measures employed in the study, i.e. they are not connected to our research questions as such.

In the Descriptive tables, you can write the SD on items in brackets and italic after the M (e.g., M(Sd) 2.32(1.37))

Replace ‘Reverse items’ with ‘Reversely scored items’ throughout the manuscript.

Reply: Done.

Results
Add the age range, apart from the M and Sd, as in minimum and maximum.

Reply: Done.

In Table 6, the significance of the standardized Beta regression coefficients should be written. Now only the statistical significance of B is written.

Reply: In the revised version we did not conduct hierarchical regression analysis, since mediation analysis is done by PROCESS in a more precise way. Standardized coefficients are not part of the regression table any more.

I would be much more interested to see the differences in the used instruments between the two educational groups then the skewness and kurtosis. So please add another table which will show the M and Sd of the used instruments separately for the SH and STEM subsamples. Even conducting an ANOVA would be appropriate.

Reply: we see your point, and that is why we added the separate descriptive statistics for SH and STEM for each instrument. Additionally, we also added intercorrelational matrix, as well as effect sizes (Cohen's d) for nominal variables. All this, we hope, should provide a clearer insight into the variables relatioinships.

Discussion
Start with a sentence that reminds the reader of the aim of the study.

Reply: Done, we start with the brief summary of the results and their relation to our research questions.

At the end, I also think it is important to state the overall limitations of an online survey, your sample and methodology in general.

Reply: Done.

Highlights
I suggest rewriting the highlights as more comprehendible or fuller sentences, it is hard for me as a reader to follow it in this form.

Reply: Done

Overall
Language and grammar require some editing (especially in the highlights section), so I suggest carefully reading through the manuscript and double checking or engaging a native speaker. I would also emphasize that the variables used here are not the only ones that are shown to be associated with vaccine conspiracy beliefs (another example from Croatia is DOI: 10.1080/08870446.2019.1673894)

Reply: Done

Reviewer #2: This article is exploring the differences in vaccination conspiracy beliefs between people belonging to different scientific fields – Social Sciences & Humanities (SH) and Science, Technology, Engineering and Mathematics (STEM). The results revealed that those educated in SH were more prone to vaccination conspiracy beliefs, and this was mediated by health beliefs; lower trust in the healthcare system, and lower science literacy.
Although the authors deal with important questions for the society today, the rationale for this specific research question is not explored enough. The theoretical background need to be more explained, followed by the importance of differentiation between SH and STEM persons. Is there any specific recommendation coming from these findings (in terms of science education)?

Reply: We added an explanation of the importance of the differentiation between SH and STEM (86-90).

Some issues remained unanswered: What kind of professions exactly, do the authors have in the groups under the umbrella of STEM and SH?

Reply: Done. We added the following sentence - As for the delineation of SH and STEM fields, the official classification of Ministry of Science and Education *of the Republic of* Croatia was used.

What are the employment statuses of the respondents? Sometimes people work in completely different fields then those from which they graduated. It would be good to discuss the relation between employment-education status of the respondents and relation of those variables to the conspiracy belief.

Reply: we completely agree with this, but unfortunately we did not measure employment status, i.e. we do not have this indicator available in the dataset.

Structure of the manuscript in some parts is not adequate for the academic paper. There is no difference between Introduction and Method. Research goals and questions should be placed in the Introduction part. Method part is missing; it should stand alone not incorporated in other parts. Instruments should be placed in the Method part.

Reply: We now clearly separated research goals and hypotheses from methods, and moved the methods into a separate section (now: Methods and instruments).

Sample information is not sufficient: we need to know when the survey was conducted, whether there was ethical approval from any institution, the length of the questionnaire. Also I would recommend reporting the sample characteristics in more details.

Reply: All the details are added now ()243-254).

Additionally, descriptive statistics are usually part of results not method part.

Reply: We agree, but in our case descriptive statistics that is presented is not connected to the research questions, but has a role of presenting the characteristics of the measurement instruments.

Endorsement of scientific knowledge and vaccine conspiracy belief should be mentioned as research question (because they are presented in the tables), and the total scores could be given separately for SH and STEM part

Reply: Done, we presented all descriptive statistics separately for SH and STEM, and also added intercorrelational matrix which sheds more light onto the relationships between the variables.

Tables are not according to APA standards.
I would also like to see empirical confirmation for the assumptions mentioned in the discussion: ...Additionally, a large portion of these differences can be explained by the mediating factors that are part of the 3C's model which, according to our claim, might be stemming from the differences in epistemic cultures, i.e. teaching patterns, cognitive approaches, research traditions, goals, behaviors, logic and methods. How can authors assume that? In the discussion part it would be better if the authors analyze results that they measured in the study.

Reply: we also agree with this, that is why in the revised version we emphasized that only science literacy was a statistically significant mediator. But we also pointed out to the fact that confidence intervals in the bootstrapping analyses of the remaining two mediators include zero effect by only a small margin, and that the coefficients go into the direction that is consistent with the assumed epsitemic differences between the science fields. In other words, we recommended that these mediators are worth researching in studies with other (random) samples.

Finally, what is the relation between Conspiracy beliefs vs. Vaccine intentions? What are the effects of conspiracy belief in the current pandemic? And how can the results of the study be connected to the situation in pandemic – covid-19 conspiracy theories. The connection between vaccine CT and Covid-19 CT should be explained.

Reply: We added the commentary of the aplicabiltiy of our results to the management of COVID-19 pandemic. In brief, we pointed out to the fact that vaccination conspiracy beliefs are strongly connected to health beliefs (importance of natural immunity) and to the trust in healthcare system (this can be seen from the intercorrelation matrix and from the results of regression analysis), i.e. that that vaccine hesitancy cannot be reduced solely by providing factual information.

Reviewer #3: The goal of the present study was to determine whether were differences in vaccine hesitancy and likelihood of accepting vaccine conspiracy theories in individuals educated in social sciences and humanities versus those educated in STEM. The research question is interesting and timely given concerns related to COVID-19 vaccine hesitancy. However, the rationale is underdeveloped, the method/results lack important details, and the discussion over-reaches. Below, I outline my concerns in more detail.

The introduction is underdeveloped. The authors mention the 3Cs at the beginning of the introduction and review some research on the “confidence” component, but no attention is given to convenience or complacency. They then state that they will examine the 3Cs in relation to those educated in STEM and social sciences/humanities. It is unclear how or why the 3Cs are being examined.

Reply: we agree, but 3Cs model was described only to point out that our mediators are not chosen at random, but that they are a part of a wider picture which can be systematized by 3Cs model. We added explanation which elements of 3Cs model we are using - institutional trust (confidence), postmodern health beliefs (complacency), and science literacy (convenience - “ability to understand”).

More discussion and connection to the research questions would be helpful. The authors also outline differences between STEM and social sciences/humanities fields, but don’t clearly connect this to vaccine hesitancy. It would be helpful for the authors to more explicitly state their reasoning and provide hypotheses, if they had a priori hypotheses. Clearer rationale for the mediation analyses needs to be provided.

Reply: we hope that we adressed these concerns in the revised version of the manuscript. We did transform or research questions into hypotheses, because we indeed posed hypotheses based on our reflection of epistemic differences between SH and STEM.

More information needs to be provided regarding participant recruitment. How were participants contacted? What was the response rate? Is the sample representative of the population in question? What range of degrees are represented in the sample (e.g., BS/BA, MS/MA, PhD, etc.)? Did participants receive compensation? Was there informed consent?

Were there any missing data, outliers, non-normality, etc.? If so, how were these issues addressed?

Reply: We added more information about sample and recruitment (243-254), as well as about ethical issues.

There were not significant outliers, while non-normality should be successfully handled by PROCESS according to Hayes book (Introduction to Mediation, Moderation, and Conditional Process Analysis, 2018, New York: Guilford Press). As for missing values, about 7,53% of all cases did have missing values on at least one of the study variables. We conduced binary logistic regression with the missing value as the criterion variable and did not find any significant relationship with other study variables.That is why we proceeded with the complete case analysis, which reduced our sample from 624 do 577 respondents. The description is now added into the revised version of the manuscript.

The research data are uploaded as the Supporting information during the paper submission process.

Why were age, gender, and religiosity included as covariates? Did results differ if covariates were excluded?

Reply: These covariates were chosen because of the possible connection to vaccine hesitancy based on the previous studies. The results did differ with regards to gender, and this was an excellent point that we now added to the discussion of the results.

Before reporting the mediation analysis, it would be useful to report simple comparisons (t-tests) indicating whether STEM vs. SH participants differed in any of the mediators or outcome variables.

Reply: This is a very good point, and it was also suggested by other reviewers. We did this by adding the intercorrelation matrix and effect sizes (Cohen's d) of the nominal variables. Hopefully, this shed more light onto the relations between the study variables before the mediation analysis.

A clearer description of the mediation analysis(es) is necessary, noting how variables were coded (i.e., STEM vs. SH) and included in the model. I assume a parallel mediation model was estimated.

Reply: Yes, it as a multiple parallel mediation. We added a conceptual diagram that should make the entire procedure much clearer than in the initial version of the manuscript.

Remove reference to marginal significance (p = .06). If relying on p-values, an effect is significant or not. Also, discussion of full or partial mediation is inappropriate, given the correlational and cross-sectional nature of the study.

Reply: Yes, we avoided this reference. But having in mind that confidence intervals include zero effect by only a small margin, form the point of view of Bayesian statistics as well for theoretical reasons (all mediation coefficients point to the hypothesized direction), we just emphasized that the suggested mediating mechanisms are worth of researching in future studies with random samples.

It is unclear to me what purpose the hierarchical regression analysis serves.

Reply: PROCESS is a regression-based method and hierarchical regression is also used to study mediation. However, PROCESS is a strict method that gives confidence intervals for direct and indirect effects, while hierarchical regression is much more „impressionistic“ method, i.e. it does not include explict test of the effects. That why we followed your suggestion and removed the hierarchical regression. As noted PROCESS is regression-based, so we provided the overall regression that is a part of the PROCESS output. This is useful because it shows the direct effects of all predictors. The other part of the story (overall and indirect effects of the field of study) is presented in the mediation analysis. Hopefully, we made it all clearer in the revised manuscript.

The claim in the discussion that the 3Cs mediated difference in education field and vaccine conspiracy attitudes is misleading as two of the three indirect effects were not significant. The discussion should be based on empirical findings from the study, not speculation of mechanisms unsupported by the data. For example, is there empirical evidence to suggest that SH individuals are more likely to endorse CAM than STEM individuals? If not, the authors need to temper their claims in the discussion.

Reply: We agree, and we made it excplicit now what the study results showed (only one of the mediation paths is statistically significant), and what are our hypotheses.

Another limitation is the correlational, cross-sectional nature of the study. You cannot truly test mediation. Be sure to avoid causal language. Alternative explanations for the findings should also be considered. There are a number of unmeasured factors that may differ between SH and STEM individuals.

Reply: we fully agree, that is why we included demographic control variables. And we are also aware that the study is correlational, we added it as an additional limitation at the end of the paper.

Minor Comments
I suggest reformatting the paper to include section headers (e.g., Method).

For all measures, indicate what direction of scores to help readers interpret the data. For example, do higher scores on the vaccine conspiracy attitudes scale indicate more or less vaccine hesitancy?

Reply: Added.
