# Supplementary material for: Vaccination conspiracy beliefs among social science & humanities and STEM educated people—An analysis of the mediation paths
Source: PLoS One. 2022 Mar 1;17(3):e0264722. doi: 10.1371/journal.pone.0264722 (PMC8887742; doi:10.1371/journal.pone.0264722)
Supplement: Attachment — Submitted filename: Response to Reviewers and Editor.docx [file pone.0264722.s005.docx]

**Editor**

Dear Editor,

Thank you for giving us the opportunity to revise and resubmit new version of this manuscript. We appreciate the time and suggestions provided by reviewer 3 and by you and we have incorporated the suggested changes into the manuscript.

We have responded specifically to each suggestion below, beginning with your own.

- throughout: change “persons” to “people”

Response: Done

- throughout: be consistent in your use of tense. Often past and present tenses are mixed, even in the same paragraph (e.g. paragraph starting line 176, paragraph starting line 281), which is very confusing for the reader.

Response: Past tense is now consistently used throughout the paper.

- 45: “become a commonplace” is hardly used in English, change to “has become commonplace”

- 56: “Various research” change to “Research” and add some references.

Response: Done.

- 62 and 486: “and healthcare system” change to “and the healthcare system”

Response: Done.

- 76: “vaccine hesitance” change to “vaccine hesitancy”

Response: Done.

- 81: add comma after “[28]”

Response: Done.

- 101: I find the term “socialization” somewhat problematic as it seems to suggest that it is an exclusively external influence (basically, all “nurture”) without allowing the fact that certain personality traits make people more or less likely to pursue a “soft” or “hard” science. Ideally, one would want to carry out a study amongst final year secondary school students who are planning to go into either direction.

Response: We agree. The term „socialization“ is changed to „background“, since the latter term covers both the academic socialization and the preexisting differences among the students. Also we added sentence „Such differences can be the result of the different academic socializations and/or the individual characteristics which are important when choosing field of study.” Also in chapter Conclusion and limitations „The reasons could probably be found in the different academic socialization and the background characteristics which are important when selecting a field of study.“

- 112: “there only few” change to “there are only few”

Response: Done.

- 113: “differences” – unclear what the directions of the differences are.

Response: The direction is now explicitly stated („mainly confirming that vaccine hesitancy is higher among SH graduates“).

- 125-129: please rewrite the sentence as I am unsure what is being stated.

Response: Done.

- 139: “on the sample” change to “on a sample”

Response: Done.

- 151-155: another difference between SH and STEM is a difference in focus, with STEM more focused on groups and averages and SH on individuals.

Response: We agree, but that topic was not covered in the research that we refer to.

- 165: willing to accepted” change to “willing to accept”

Response: Done.

- 204: you have to start with the participant info otherwise the results in Tables 1 - 4 are uninterpretable.

Response: The participants' info is moved to the beginning of the chapter.

- as suggested by one of the Reviewers in the previous round, please add simple t-tests when splitting the data between groups, e.g. lines 209-210).

Response: Done.

- 210, 220, 233: “higher results” change to “higher values”

Response: Done.

- Table headings for Tables 1 to 4: “for THE… scale”

Response: Done.

- 237: please add a reference for the Oxford scale

Response: Done.

- 249: please add references for the surveys referred to.

Response: Done.

- 266: “consisted of the college”: delete “the”

Response: Done.

- 269: “conduced” change to “conducted”

Response: Done.

- 277: add a reference or link for the classification used

Response: Done.

- 303: delete “Andrew F.”

Response: Done.

- Tables 6 & 7: define LLCI and ULCI

Response: Explanations added.

- paragraph starting 308: why are the numbers (e.g. 1.36) different from those in the Table?

Response: It is corrected now.

- 315: “is statistically” change to “is a statistically”

Response: Done.

- 337: “significant”: where is this indicated in the Table?

Response: It is visible since the confidence interval includes zero (effect).

- 352: “with the other” change to “with other”

Response: Done.

- 372: “additional analyses”: where can the reader find these?

Response: We included them in the paper now (i.e. the magnitude of the indirect effects when gender is not a covariate). All analyses can be easily replicated by using our data and SPSS PROCESS syntax (process y=Vac_con /x=Stu_re /m=Lit Imm HCS /cov= Age Relig Gender /model=4.) Since PROCESS uses bootstraping results can be negligibly different from analysis to analysis, but the conclusions are always the same (we repeated all analyses several times).

- 382: delete “so”

Response: Done.

- 385-386: “sample of…”: please rephrase

Response: It is rephrased as: „as well as with a research conducted on a sample of Croatian population”.

- 428: “found…correlation”: change to either “found a ... correlation” or “found… correlations”

Response: Done.

- 430: “indicate” change to “indicates”

Response: Done.

- 432: “having in mind that” change to “in that”

Response: Done.

- 432: “the vaccine” change to “vaccine”

Response: Done.

- 455: “to much” change to “too much”

Response: Done.

- 456: “object the fact” change to “object to the fact”

Response: Done.

- 471: “as well with” change to “as well as with”

Response: Done.

- 494: delete “in it”

Response: Done.

Just as a suggestion, I think some of the research by Gordon Pennycook and colleagues will be of interest to your future research, e.g. their examination of a bias to accept “pseudo-profound bullshit”, which is linked with e.g. religious beliefs and analytical thinking (e.g. Pennycook & Rand, 2019; DOI: 10.1111/jopy.12476).

Response: Than your for the suggestion, we are going to use it in the continuation of our project.

**Reviewer #3:**

Reviewer #3: Overall, the authors have done a nice job addressing my initial concerns and suggestions. The manuscript is much stronger. I do have a few minor suggestions.

1. In the introduction, it would be helpful to explicitly connect field of study with the 3Cs to clearly articulate rationale.

Response: the following sentence is added: „. Additionally, it is our contention that such differences will be mediated by some of the indicators coming from the 3Cs model (trust in the healthcare system, specific health beliefs, and science literacy).”

2. There are still a few places in the manuscript where the authors refer to "causal" relationships. That cannot be determined from this study.

Response: We checked it, we use this word only in the following sentence: „In addition, given that our study was cross-sectional and the possible omitted variables bias might have existed, causal mechanisms cannot be fully supported by the data from our study.“. Following suggestions of the reviewer, in other parts of the paper we only refer to „correlations“, „connections“, etc. Please let us know if we are wrong, i.e. if we missed some of the causal implying language.

3. Provide an explicit rationale for the inclusion of covariates in the analyses. Also, as the some of the results were different when covariates were excluded, report the stats in text or supporting material.

Response: The following sentence is added: „. The covariates were used in order to check for the possible spurious effects of our predictors.”.

The stats (indirect effects) of the model when gender is not added as a covariate are now reported in the text.

4. It is important to acknowledge that differences between education and training in these fields may not be the reason for these findings. Rather, there may be unmeasured individual differences (e.g., personality, cognitive styles, etc.) that lead people into different educational fields and influence vaccination conspiracy beliefs.

Response: Yes, we agree. That is why we replaced the term „academic socialization“ with „academic background“, since the latter term implies both possible explanations. The following sentence is also added: „Such differences can be the result of the different academic socializations and/or the individual characteristics which are important when choosing a field of study.”(in Introduction) and „The reasons could probably be found in the different academic socialization and the background characteristics which are important when selecting a field of study.“ (in chapter Conclusion and limitations).
